# Performance and Mechanism Analysis of an Anti-Skid Wear Layer of Active Slow-Release Ice–Snow Melting Modified by Gels

**DOI:** 10.3390/gels11060449

**Published:** 2025-06-11

**Authors:** Yuanzhao Chen, Zhenxia Li, Tengteng Guo, Chenze Fang, Peng Guo, Chaohui Wang, Bing Bai, Weiguang Zhang, Haobo Yan, Qi Chen

**Affiliations:** 1 School of Civil Engineering and Transportation, North China University of Water Resources and Electric Power, Zhengzhou 450045, China; zhenxiali2009@ncwu.edu.cn (Z.L.); guotth@ncwu.edu.cn (T.G.); fangchenze@126.com (C.F.); baibing7777@ncwu.edu.cn (B.B.); 15737993622@163.com (H.Y.); chq319666@163.com (Q.C.); 2 Henan Province Engineering Technology Research Center of Environment Friendly and High-Performance Pavement Materials, Zhengzhou 450045, China; 3 Technology Innovation Center of Henan Transport Industry of Utilization of Solid Waste Resources in Traffic Engineering, North China University of Water Resources and Electric Power, Zhengzhou 450045, China; 4 Henan Province University-Enterprise Research and Development Center for Green, Low-Carbon and High-Performance Road Materials, Zhengzhou 450045, China; 5 National and Local Joint Engineering Laboratory of Traffic Civil Engineering Materials, Chongqing Jiaotong University, Chongqing 400074, China; guopeng@cqjtu.edu.cn; 6 School of Highway, Chang’an University, Xi’an 710064, China; wchh0205@chd.edu.cn; 7 School of Transportation, Southeast University, Nanjing 210096, China; wgzhang@seu.edu.cn

**Keywords:** pavement materials, slow-release de-icing material, microscopic, characterization, cold-climate pavement, ceramsite, road performance, slow-release mechanism, gel material

## Abstract

Winter pavement maintenance faces challenges in balancing large-scale upkeep and driving safety, particularly regarding the application of active slow-release materials. This study proposes a gel-modified salt-storing ceramsite asphalt mixture to enhance ice-melting capabilities through controlled salt release. By replacing a conventional coarse aggregate with salt-storing ceramsite in SMA-10 graded mixtures (0–80% content), we systematically evaluate its mechanical performance and de-icing functionality. The experimental results demonstrate that 40% salt-storing ceramsite content optimizes high-temperature stability while maintaining acceptable low-temperature performance and water resistance. Microstructural analysis reveals that silicone–acrylic emulsion forms a hydrophobic film on ceramsite surfaces, enabling uniform salt distribution and sustained release. The optimal 10% gel modification achieves effective salt retention and controlled release through pore-structure regulation. These findings establish a 40–60% salt-storing ceramsite content range as the practical range for winter pavement applications, offering insights into the design of durable snow-melting asphalt surfaces.

## 1. Introduction

With an increasing amount of road traffic, road surfacees will exhibit the phenomenon of tectonic depth attenuation and flatness reduction [1]. At present, the treatment methods of China’s highway maintenance include washing and planing the original road surface to lay a new asphalt surface layer, micro-surface or thin slurry sealing layer, and paving anti-skid wear layer [2]. When an anti-skid wear layer is applied to a road surface, damage to the structural layer of the road surface is reduced, improving the performance of the surface and extending its service life [3]. When the paving thickness is small, usually in the range of 15~25 mm, it can reduce the consumption of materials and energy, reduce initial costs and maintenance costs, and is an environmentally friendly road paving technology, in-line with the concept of global environmental protection.

At present, in most of the northern regions of China in winter, snow and ice phenomena appear on road surfaces in rainy and snowy weather, resulting in a reduced friction coefficient of automobile tires, resulting in vehicle slippage and braking difficulties, which can easily lead to loss of control of vehicles and, therefore, traffic accidents. An active slow-release type of snow- and ice-melting material combined with asphalt pavement pre-conservation technology, salt-storing pavement de-icing, and snow-removal technology are needed, based on an innovative anti-slip wear layer with excellent anti-skid and wear-resistant performance [4], in order to prepare asphalt pavement for active pre-maintenance and achieve the effect of actively melting ice and snow on road surfaces. This can not only serve a winter pavement snow-melting and de-icing function, but also can effectively reduce the adverse effects of salt-storing snowmelt on asphalt pavements. Therefore, it is of great significance to develop pavement materials with snow-melting and ice-melting effects and maintenance functions.

Ice- and snow-melting salt material is coated on the salt-storing carrier using a modification process based on slow-release gel technology, prepared as a powder or granular composite materials [5], that can replace the aggregate and mineral powder in the asphalt mixture [6]. In recent years, experts have introduced wear layer technology into ice- and snow-melting pavements, and the results have proved that the developed materials have certain ice- and snow-melting effects. Therefore, in this paper, through the development of a slow-release composite snow-melting agent, attached to the pores of a porous aggregate, combined with anti-skid wear layer maintenance technology and salt-storing aggregate-replacing parts of aggregates of the same particle size, we study the ice- and snow-melting performance of a road treated with an active slow-release snow-melting anti-skid wear layer so as to achieve an active and lasting snow- and ice-melting function from the pavement. This is of positive significance for the coordinated and sustainable development of our society, economy, and environment, as well as for the promotion of the high-quality development of transportation engineering. Anti-skid wearing layer technology research first began in France, and scholars worldwide have proposed a thin asphalt concrete surface layer, ultra-thin asphalt concrete (UTAC), a Novachip anti-skid wearing layer based on synchronized paving technology, a Novachip ultra-thin wearing layer, and an open-graded anti-skid wearing layer (OGFC). On the basis of previous research, American scholars successively launched SUP-5 and SMA-5, two more advanced fine-grained ultra-thin wearing layer technologies. In recent years, domestic and foreign ultra-thin wearing course technology has developed rapidly, with different types and functions of thin-layer technology emerging frequently, such as high-toughness ultra-thin asphalt wearing course [7,8,9], SMC warm-mixed thin layer [10], ECA easy-to-consolidate ultra-thin wearing course [11], temperature-controlled ultra-thin wearing course [12], as well as active-melting snow and ice ultra-thin wearing course, etc. At present, active-melting snow and ice anti-skid layer is the most advanced thin-layer technology. At present, the active ice- and snow-melting anti-skid wear layer is divided into salt-storing ice- and snow-melting anti-skid wear layer and energy-conversion anti-skid wear layer according to the principle of melting ice and snow. Energy-transforming anti-skid wear layer melting ice and snow consumes a lot of energy, and the energy and heat inevitably affect the pavement structure [13,14], which can be avoided using a salt-storage melting ice and snow anti-skid wear layer. Ice- and snow-melting materials are combined in some form (filler or aggregate) with ultra-thin wear layer materials, thus introducing three new influencing factors, i.e., changes in the oil/gravel ratio due to differences in filler properties [15,16], changes in the mix structure due to particle interferences [17,18], and changes in the voids caused by the salt that is removed [19,20]. Based on the above problems, researchers have carried out related research work. For example, Sun Dahang [21] proposed a new evaluation index and design method for an ice-suppressing anti-skid wear layer; Liu Yang [22] investigated the impact of slow-release ice-melting and snow-melting material doping on the road performance of ultra-thin wear layer by using single-factor analysis and constructed an optimal custom model to optimize the composition of the material based on the Response Surface Analysis method; Chen Shuanfa [23] obtained the basic composition and key design parameters of the material from three aspects, namely, the composition of the mineral grades, asphalt paste characteristics, and road performance of TSSAM, and verified the basic material composition and long-lasting snow-melting performance and road performance of TSSAM under different conditions. Chen Shuanfa [23], regarding the TSSAM mineral gradation composition, asphalt slurry characteristics, and road performance, researched the basic composition of the material and the key design parameters, and verified snow-melting performance and snow-melting long-lasting performance under different conditions; Chen Xiao [24] used SMA-5 gradation as an ultra-thin snow melting cover, and, through the study of road performance and snow-melting and ice suppression function, concluded that, when the mixing amount of the snow-melting agent is 33%, the ultra-thin snow-melting covers with various road performance and snow-melting performance all meet the requirements. The road performance and snow- and ice-melting performance of the ultra-thin snow-melting cover meet the requirements.

Comprehensive research has shown that an active slow-release ice-melting snow anti-skid wear layer, compared to other salt-storing pavement de-icing and snow-removal technology [25], can improve asphalt pavement skid resistance and abrasion resistance, can be considered to extend the service life of the pavement, and represents a new technology for snow-melting and de-icing, offering better road performance. However, due to the lack of actual engineering experience, design specifications, and evaluation standards, active slow-release ice-melting and snow-melting anti-skid wear layer pre-maintenance technology is still in its infancy. How to prepare slow-release composite snow-melting agents, combined with anti-skid wear layer maintenance technology, salt-storing aggregates [26,27,28,29] as a partial replacement for aggregates of the same particle size, the study of active slow-release ice-melting and snow-melting [30,31,32,33], and the performance of the snow-melting wear layer, as well as snow- and ice-melting performance, in order to achieve active and durable road melting and snow-melting functions, is an important issue to be solved at present [34,35,36]. Function is an important problem to be solved at present. Many scholars have performed research on the anti-slip performance of the anti-skid wear layer, surface functional layer performance, and snow-melting on pavements, mainly including the anti-skid performance of wear layers, surface functional layer performance, and a series of research and development work on snow-melting technology on pavements; however, in the current literature, few studies have addressed snow-melting performance and the mechanisms of active slow-release anti-ice wear layers. This paper, therefore, investigates the performance and underlying mechanisms of gel-modified salt-storing ceramsite in anti-skid wearing layers, addressing this identified gap in knowledge. Compared to traditional de-icing methods (e.g., bulk salt application or heated pavements), our gel-modified salt-storing ceramsite approach has comparable initial costs, but significantly lower long-term maintenance requirements, and its slow-release functionality extends the economic service life of pavements. This paper explores the advantages of anti-skid wear layers with environmentally friendly slow-release snow- and ice-melting functions.

## 2. Results and Discussion

### 2.1. Road Performance Test Results

**Figure 1:**
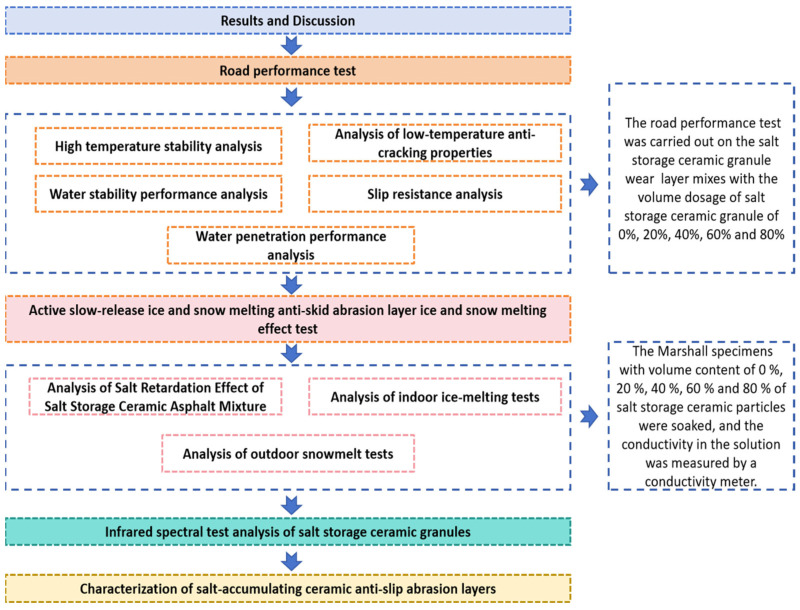
Technology roadmap.

Referring to JTG E20-2011 [37], the road performance test was carried out on the salt-storing ceramic granule wear layer mixes with volume dosages of salt-storing ceramic granules of 0%, 20%, 40%, 60% and 80%, respectively, and the effect of different slow-release salt-storing ceramic granule dosages on the road performance of the active ice-melting and anti-skid wear layer was analyzed by combining the results of the tests in each group. The experimental research plan is shown in Figure 1.

#### 2.1.1. High-Temperature Stability Analysis

**Figure 2:**
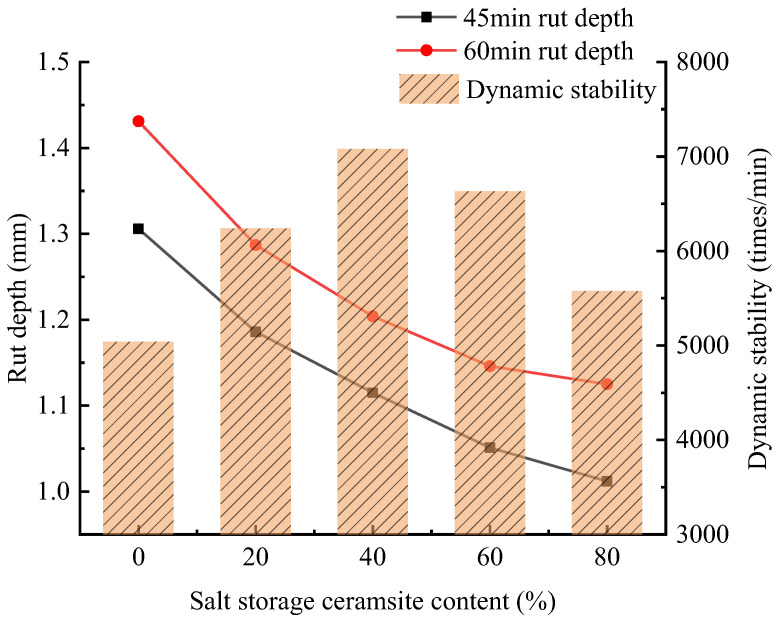
Wheel-tracking test results.

The rutting test was used to evaluate the high-temperature stability. Five rutted plate specimens doped with salt-accumulated ceramic grains were molded, and the rutting test was carried out by holding the heat for 4 h at 60 °C; the rutting results of different salt-accumulated ceramic grain-doped specimens are shown in Figure 2.

As can be seen in Figure 2, the dynamic stability of the anti-slip wear layer mix under different salt-storing ceramic granule dosages meets the specification requirements. The dynamic stability of the wear layer mix with the increase in salt-storing ceramic particles showed a trend of increasing and then decreasing; when the dosage of salt-storing ceramic particles was 40%, the dynamic stability of the wear layer mix reached the maximum, and its dynamic stability increased by 40.4% compared to the mix without salt-storing ceramic particles. It can be concluded from the test results that more salt-storage ceramic particles does not necessarily mean the better the high-temperature stability of the mixture; as far as high-temperature stability is concerned, the optimal proportion of salt-storage ceramic particles is 40%. These results align with the findings of Chen Xiao et al. [24], who reported an optimal snow-melting agent content (~33%) for an ultra-thin asphalt layer, indicating consistency across studies. Salt-storing ceramic particles improve the high-temperature stability of the wear layer mixture: (1) Slow-release salt-storing ceramic particles and polymer-modified cement mortar coat the salt-storing ceramic particles due to the alkaline cement, so that modified cement mortar parcel salt-storing ceramic particles and asphalt have good adhesion; adding salt-storing ceramic particles after polymer-modified cement mortar parcel means that its strength improves and that it shows better wear resistance, and so the mixture is good. The strength of salt-accumulating ceramic granules is improved after the polymer-modified cement mortar is wrapped, and the abrasion resistance is better, so the high-temperature stability of the mix is good. (2) Shale grains are mainly shale materials, as raw materials calcined at high temperatures have certain activity on their surfaces, so that, as the contact surface strength of the grains and asphalt increases, the friction of the wear layer mixture increases; the salt-storing grains of the ceramic anti-skid wear layer with high-temperature stability have a certain effect of enhancement. (3) Due to the structural characteristics of ceramic grains, the surface is rough and there are a large number of pore structures, and the salt storage ceramic grains are larger than the ordinary crushed stone. The effective surface area of the aggregate is large, so that, as the bonding area between the asphalt and the salt grains increases, its adsorbs effective asphalt and also adsorbs excess asphalt, improving the internal cohesion of the mixture, which in turn increases the shear resistance of the salt-grain anti-skid wear layer.

#### 2.1.2. Analysis of Low-Temperature Anti-Cracking Properties

**Figure 3:**
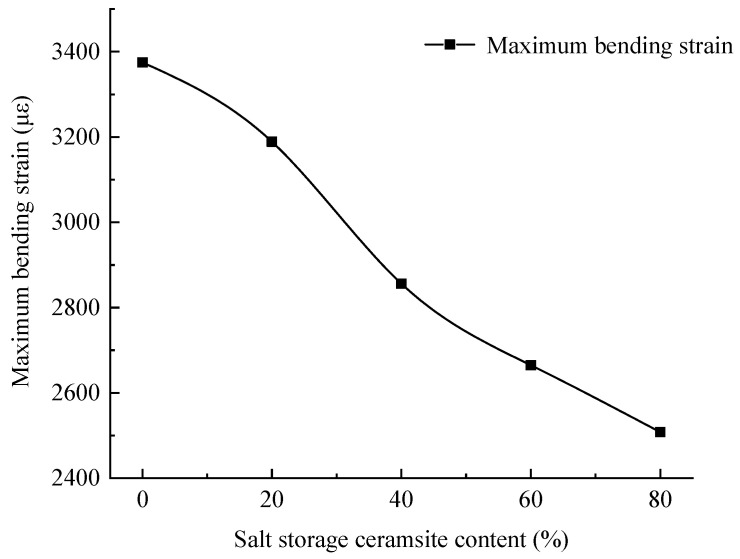
Test results of the maximum bending strain of trabecular.

The low-temperature trabecular bending test was used to evaluate the low-temperature performance of salt-storing ceramic grain wear layer mixtures which were placed in a constant temperature box at −10 ± 0.5 °C for 5 h and loaded with a universal testing machine at a constant rate until the destruction of the specimen; the results of the low-temperature trabecular bending test with different salt-storing ceramic grain admixtures are shown in Figure 3.

As can be seen in Figure 3, the maximum bending strain and flexural tensile strength of the salt-storing ceramic grain abraded layer mixtures showed different degrees of reduction with increases in salt-storing ceramic grain dosage, which indicates that the salt-accumulated ceramic grain dosage has a greater impact on their low-temperature performance. At 20%, 40%, 60%, and 80% of the volume of salt-storing grains, the flexural tensile strength of the wear layer mix was reduced by 14.3%, 27.3%, 35.0%, and 41.9%, respectively, and the maximum flexural strain was reduced by 5.5%, 15.4%, 21.0%, and 25.7%, respectively, compared to that of the control mix without salt-storing grains. The main reasons are as follows: (1) Due to the crushed-stone shale grains after the addition of the polymer-modified cement mortar package and after the angularity becomes poor, the salt-storing grains in the anti-skid wear layer mixture cannot form a strong embedded structure, resulting in insufficient internal friction, which causes the wear layer mixture with salt-storing grains to deform easily under external force. (2) Due to the low strength of the salt-accumulated ceramic particles, the ceramic particles become brittle after freezing at −10 °C, the overall resistance of the specimen becomes insufficient, and the ceramic particles fracture earlier than common gravel; therefore, the low-temperature performance will deteriorate.

#### 2.1.3. Water Stability Performance Analysis

Water stability was evaluated using the immersion Marshall Test and the freeze–thaw splitting test. The results are as follows.

##### Immersion Marshall Test

**Figure 4:**
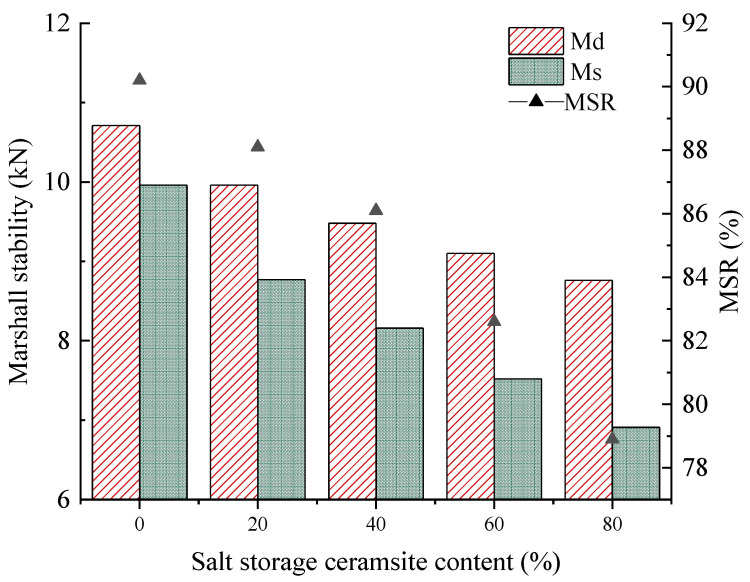
Immersion Marshall test results.

Five different dosages of salt-storing ceramic asphalt mixtures were used to mold two groups of Marshall specimens, each containing four specimens: Group 1 was immersed in a constant-temperature water tank at 60 °C for 30 min to test stability; and Group 2 was immersed at 60 °C for 48 h to test stability by calculating the residual stability of the specimens to evaluate the water stability of different dosages of salt-storing ceramic asphalt mixes. The results of the immersion Marshall test with different salt-storing ceramic grain dosages are shown in Figure 4.

As can be seen from Figure 4, 20%, 40%, and 60% dosages of salt-storing ceramic grain wear layer mixture residual stability meet the MSR ≥ 80% specification requirements. Compared to the 0% dosage of salt-storing ceramic grains asphalt mixture, the addition of salt-storing ceramic grains reduces the residual stability of the wear layer mix. The higher the dosage of salt-storing ceramic grains, the lower the residual stability of the Marshall specimens. This is mainly due to the structural characteristics of the ceramic grains; salt-storing ceramic grains, compared to ordinary aggregate strength, are relatively insufficient and have a high water absorption rate. Therefore, with more ceramic particles mixed, water in the asphalt mixture damage is more significant, and the worse the water stability is. Doping with amounts of 20%, 40%, and 60% of salt-storing ceramic grains meants that residual stability, compared to the ordinary asphalt mixture, decreased by 2.3%, 4.5%, and 8.4%; when the salt-storing ceramic grain dosage increased to 80%, the residual stability of the wear layer mixture no longer met the specification requirements. This means that a certain amount of slow-release salt-storing ceramic particles reduces the water stability of the wear layer asphalt mixture; the dosage is too high, meaning that the residual stability does not meet specification requirements.

##### Freeze–Thaw Splitting Test

**Figure 5:**
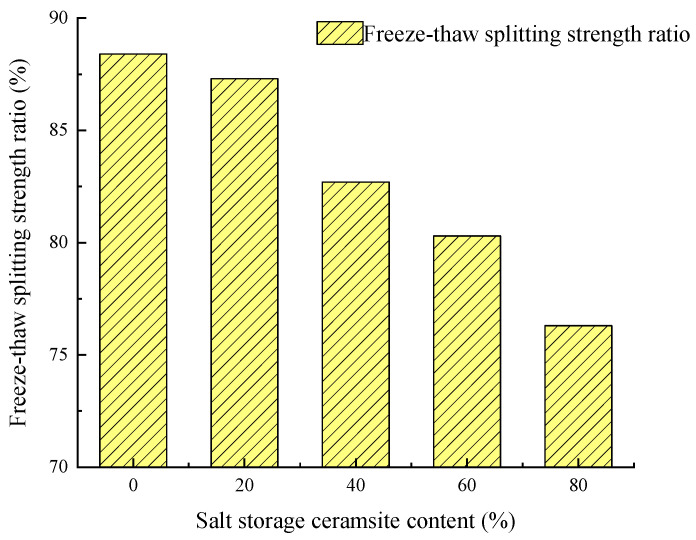
Freeze–thaw splitting strength ratio test results.

We referred to T 0729 in JTG E20-2011 [37] to perform the execute the freeze–thaw splitting test. The results of the freeze–thaw splitting test with different salt-storing ceramic granule dosages are shown in Figure 5.

The larger the value of the split strength ratio, the stronger the mix’s resistance to water damage. In Figure 5, it can be seen that the split strength ratio decreases gradually with the increase in salt-storing ceramic granule doping, and the split strength of 20%, 40%, and 60% salt-storing ceramic granule-doped wear layer mixes was reduced by 1.2%, 6.4%, and 9.2% compared with that of the ordinary wear layer mix. In the test process, it was found that, after the freezing and thawing of the Marshall specimen, a particle spalling phenomenon could be seen, and, as the salt-storing ceramic granule dosage increased from 20% to 80%, the spalling phenomenon became more severe; when the dosage reaches 80%, the splitting strength of the mixture no longer meets the specification requirements.

In the test results, it can be seen that the salt-storing ceramic particles mixed into the wear layer mixture have a significant impact on water stability. There are two main reasons for this: First of all, asphalt and ceramic particles on the surface of the combination are weak, resulting in low cohesion within the mixture; in the role of the dynamic hydraulic force, large-scale peeling of the asphalt overlay layer may occur, which affects the water stability of the salt-storing ceramic particle wear layer. Secondly, the structures of the salt-storing ceramic particles and of the specific salt grains themselves are also an important factor due to the high water absorption rate and the strong water retention, meaning that water can enter the mixture easily and is difficult to dissipate; therefore, water absorption and retention are significant characteristics, and, under vehicle loading, the mix becomes loose, which causes the salt grain wear layer mixture to become easily damaged under the action of external forces or the freeze–thaw cycle.

According to the salt grains wear layer mixture of the immersion Marshall test and freeze–thaw ratio test, it can be seen that, at 80% salt grain dosage, the Marshall stability of the mixture and split ratio do not meet the requirements; at 60% dosage, the split strength ratio is 80.2%, which just meets the specification requirements. In order to ensure that the salt-storing ceramic wear layer mixture has good water stability, the salt-storing ceramic mix dosage should be less than 60%.

#### 2.1.4. Slip-Resistance Analysis

Anti-sliding performance was evaluated using the sanding method and pendulum instrument. The results are as follows.

##### Sand-Laying Method

We used the sand-laying method to determine the construction depth of different salt-storing ceramic granule dosages of ice- and snow-melting anti-skid wear layer mixture; the test results are shown in Table 1 below.

As can be seen in Table 1, the construction depth of asphalt mixtures with five different ceramic granule dosages satisfies the technical requirements of the specification (TD ≥ 0.55 mm). With the increase in salt-storing ceramic particles, the pavement diameter gradually becomes larger, the construction depth gradually decreases, and the rutting depth of the rutting plate with 20%, 40%, 60%, and 80% salt-storing ceramic particle dosages decreases by 3.1%, 5.0%, 8.6%, and 9.7%, respectively, compared to that of the rutting plate with 0% salt-storing ceramic particles. This is due to the poor angularity of the salt-accumulated ceramic particles after wrapping with silica–propylene emulsion-modified cement mortar, but the effect of salt-accumulated ceramic particles on the depth of the structure is not significant, and the reduction in the depth of the structure is less than 10%. This is due to the use of grading type SMA-10, which is an intermittent grading and skeleton-dense structure, which compacts the surface of the structural depth with a large and good anti-skid performance, so the ceramic particles do not affect the anti-skid performance of the pavement.

##### Pendulum Instrument

**Figure 6:**
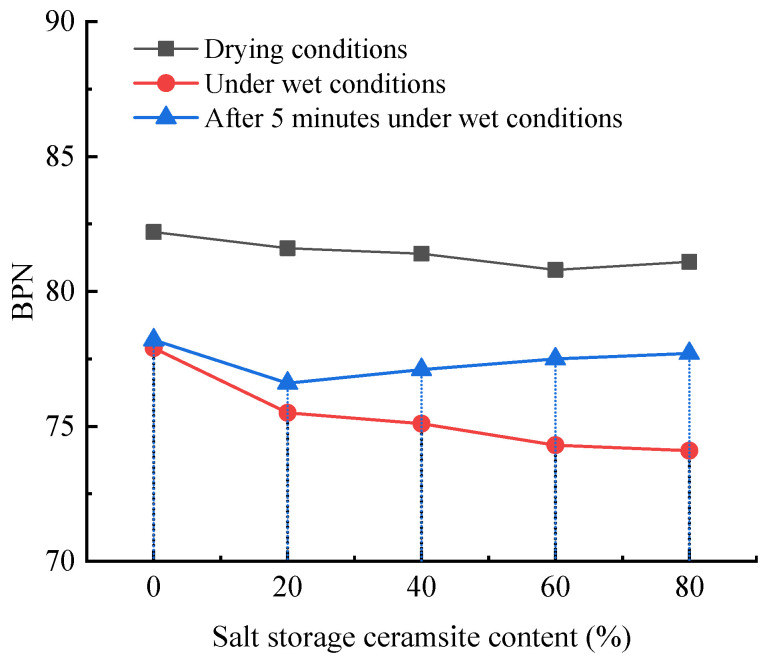
Anti-sliding performance test results.

The anti-skid performance of a slow-release salt-storing ceramic asphalt mixture rutting plate was studied using a BM-III type pendulum friction coefficient tester, and the test results are shown in Figure 6.

As can be seen in Figure 6, under dry conditions, the addition of ceramic granules results in a slight decrease in the BPN value of rutted slabs, which is almost negligible and does not affect the pavement’s skid resistance. Under wet conditions, the BPN values of all test groups decreased to a certain extent, and the BPN values of rutted slabs with 0%, 20%, 40%, 60%, and 80% salt-storing ceramic granule dosages decreased by 5.2%, 7.4%, 7.7%, 8.0%, and 8.6%, respectively, under wet conditions, and the BPN value of rutted slabs under wet conditions decreased by a larger and larger rate with the increase in the dosage of ceramic granules. This is due to the fact that the ceramic granules become less angular after being wrapped in cement mortar, and the anti-slip performance is weakened under wet conditions with the increase in ceramic granule doping. After 5 min, the wetted rutted plate was tested, and the BPN value of the rutted plate recovered to some extent, and, compared to the BPN under the wetting condition, the BPN values of rutted slabs tiwht 0%, 20%, 40%, 60%, and 80% salt-storing ceramic granule dosages increased by 0.4%, 1.5%, 2.7%, 4.3%, and 4.9%, respectively, which was due to the fact that the ceramic granules have a porous structure and high water absorption and, after wrapping the cement net mortar, the cement will also absorb part of the water so that the water dissipation of the rutted slab doped with salt-storing ceramic granules is faster than that of the rutted slab not doped with salt-storing ceramic granules, so that the BPN value increases more rapidly and the anti-skid performance is partially restored. According to this comprehensive analysis, the addition of salt-storing ceramic granules will not affect the anti-skid performance of the pavement.

#### 2.1.5. Water-Penetration Performance Analysis

Water seepage performance was evaluated using a water seepage test.

The results of percolation tests with different salt-storing ceramic granule dosages are shown in Table 2.

The results in Table 2 show that, with the increase in salt-storing ceramic granule doping, the water permeability coefficient gradually became larger; this is because the ceramic granule itself is highly absorbent and, after the cement mortar package, the cement also absorbs part of the water. The water permeability coefficient of the SMA-10 anti-slip wear layer is below the specification value of 80 mL/min, which indicates that the salt-storing ceramic granule will not have an effect on the salt-storing ceramic granule wear layer of the mixing material’s seepage performance.

### 2.2. Active Slow-Release Ice and Snow-Melting Anti-Skid Wear Layer Ice in the Snow-Melting Effect Test

The salt-storing ceramic asphalt mixture conductivity test, indoor ice-melting test, and outdoor snow-melting test were performed to analyze the different dosages of salt-storing ceramic asphalt mixtures’ snow- and ice-melting performance, combined with the road performance, to determine the optimal dosage of salt-storing ceramic.

#### 2.2.1. Analysis of the Salt-Retardation Effect of Salt-Storing Ceramic Asphalt Mixture

**Figure 7:**
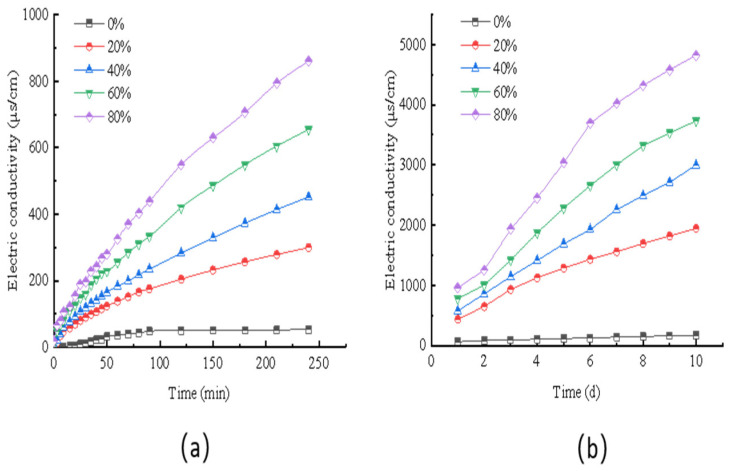
Short-term (**a**) and long-term (**b**) conductivity of salt-storing ceramide asphalt mixture.

In this section, Marshall specimens are soaked, and the conductivity of the solutions are measured using a conductivity meter to test the salt-release pattern of asphalt mixtures with different volumetric dosages of salt-storing ceramic grains and the long-term effectiveness of the salt release of the slow-release storage melting ice and snow anti-skid wear layer. The results of short-term conductivity for different salt-storing ceramic granule dosages are shown in Figure 7a, and the long-term conductivity is shown in Figure 7b.

As can be seen from Figure 7, the salt-storing ceramic particles mixed with 20%, 40%, 60%, and 80% of the mixture, compared to the ordinary asphalt mixture of snow-melting salt, shows significant synthesis, indicating that the presence of salt-storing ceramic particles in the asphalt mixture does not hinder salt precipitation, which also proves that the salt-storing ceramic particles in the asphalt mixture can melt ice and snow. From the short-term conductivity graph of the mixture in Figure 7a, it can be seen that, with an increase in salt-storing granules, the conductivity in the solution gradually increases—that is, the concentration of the snow-melting salt solution increases. The larger the dosage of salt-storing ceramic granules, the more snow-melting salt is precipitated per unit of time and the more significant the melting effect on snow and ice is. In Figure 7b, a long-term conductivity graph of the mixture can be seen; even when using different amounts of salt-storing ceramic asphalt mixture after 7 days, the conductivity can still ensure that there is a faster rate of growth. This shows that the salt in the mixture is more sufficient and can ensure the long-lasting effect of snow-melting in the actual snow-melting process.

#### 2.2.2. Analysis of Indoor Ice-Melting Tests

**Figure 8:**
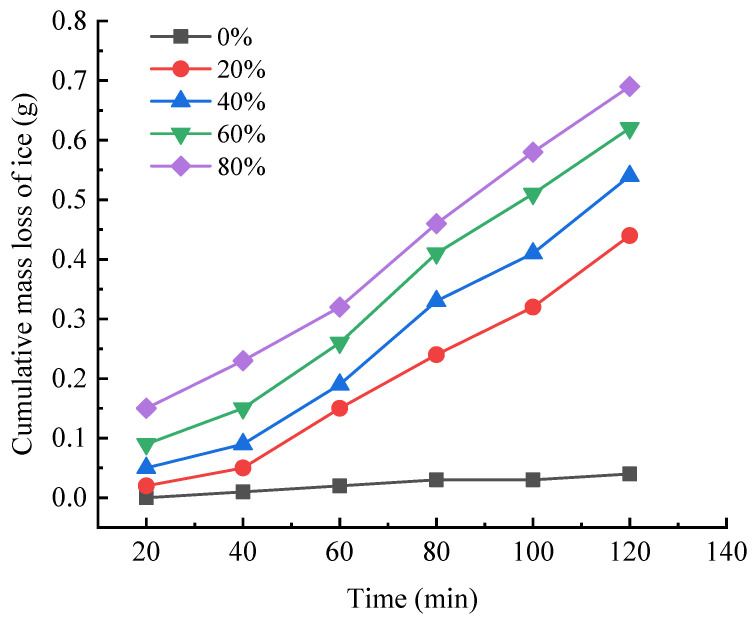
Melting mass loss trend of ice cubes.

Referring to the ‘Ice and Snow Melting Material for Highway Asphalt Mixture’ (JT/T 1210.2-2018) [38] for the ice-melting rate test, the results of the indoor ice-melting test with different salt-storing ceramic granule dosages are shown in Figure 8.

As can be seen from Figure 8, under the condition of equal ice contact, with the increase in salt-storing ceramic granules, the mass loss in the ice cubes in the same period of time shows a gradual increasing trend. The amount of ice-melting in the ordinary asphalt mixture (0% salt-storing ceramic granules) is much smaller than the amount of ice-melting in the asphalt mixture with salt-storing ceramic granules. In salt-storing ceramic particles mixed at 20%, 40%, 60%, and 80%, the amount of ice-melting after 120 min, compared to the ordinary asphalt mixture’s ice-melting, increased by 10, 12.5, 14.5, 16.25 times; therefore, it can be concluded that salt-storing ceramic particles mixed with asphalt mixture have a better ability to melt ice. Relative to the replacement of the mineral-powder-type snow-melting salt asphalt mixture, the salt-storing ceramic granule replacement of ordinary ice-melting aggregates is not very obvious; in all five groups, the ice-melting amounts were within 1 g, thus appearing to show a relatively low de-icing effect. Taking into account the environment of the box temperature of −5 °C, the loss in mass of ice is relatively small, considering the effect of vehicle load on the road surface, and more salt will seep into the water, further enhancing the melting of ice and snow. Considering that more salt will seep out with water under the effect of vehicle load on the road surface to further enhance the effect of ice- and snow-melting, the results of road use will differ.

#### 2.2.3. Analysis of Outdoor Snow-Melting Tests

**Figure 9:**
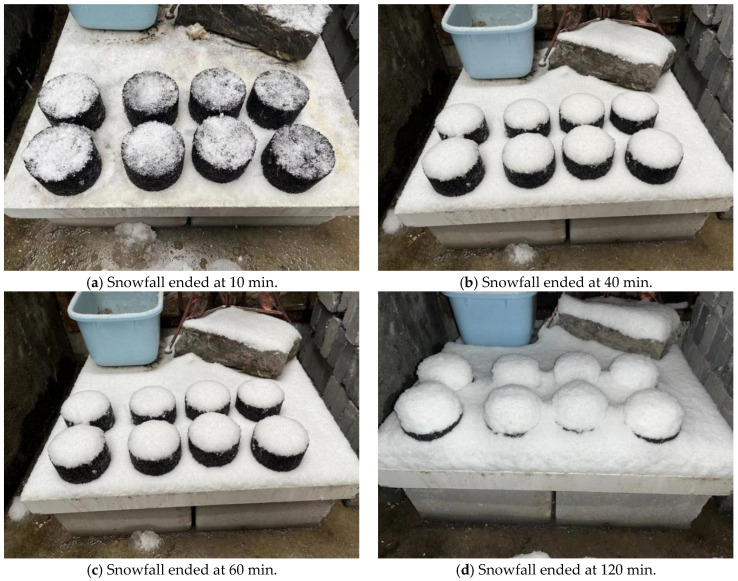
Changes in test pieces during snowfall.

Marshall specimens, from left to right, were doped with salt-storing ceramic granules in the order of 0, 20%, 40%, 60%, and 80%; the outdoor snow-melting test snowfall process affecting the specimens’ snow accumulation are shown in Figure 9.

From Figure 9a–d, it can be seen, during snowfall, that snow accumulation increased on all five specimens with different dosages. At the same time, different salt-storage dosages of specimens in the snow comparison test found that different specimens of the different snowmelt showed subtle differences in the effect of snow-melting; this is because, if the snowfall amount on that day was too high, the rate of snowfall was greater than the rate of snow melting. In this paper, the salt-storing ceramic granules were used to replace aggregates in the asphalt mixture; this type of aggregate-substituted salt-storing asphalt mixture, compared to mineral-powder-substituted snow-melting asphalt mixture, had a slower snow-melting rate in the first period. Therefore, in this paper, regarding snowfall, different salt-storing ceramic grain dosages of snow retention on the test specimen were observed, compared, and analyzed to examine the snow-melting effects of asphalt mixtures with different dosages of salt-storing ceramic granules.

**Figure 10:**
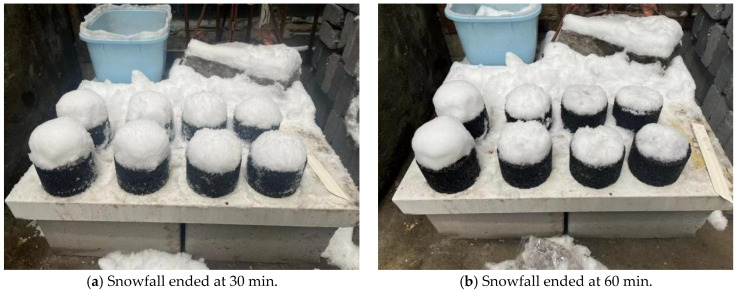

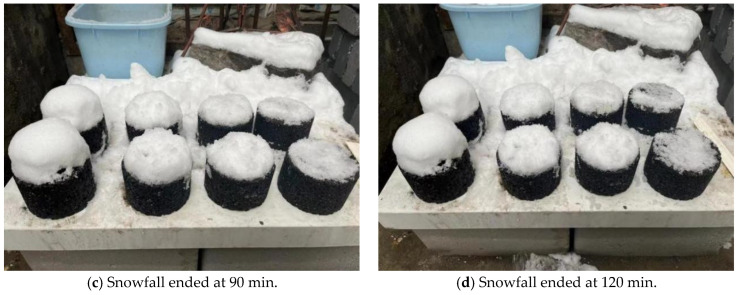
Snow-melting test.

The snow melting on top of the Marshall specimens at various times after snowfall is shown in Figure 10.

From Figure 10a–d, it can be seen, from left to right, that, with the increase in salt-storing ceramic granule mixing, the amount of snow on the specimen gradually decreases in the 20% dosage of the specimen, with a subtle effect of melting snow; the 60% and 80% dosage specimens’ melting snow effect is more significant. As shown in Figure 10, image analysis at t = 120 min demonstrates that the sample with 80% salt-storing ceramsite content exhibits approximately 5% residual snow cover (indicating nearly complete melting), whereas the sample with 20% content retains approximately 80% snow cover. This contrast highlights that higher salt-storing ceramsite content is correlated with enhanced snow-melting capacity. Consequently, the snow-melting rate demonstrates a clear positive correlation with increasing salt-storing ceramsite content, reflecting a significant acceleration in melting efficiency as content rises. In the salt-storing ceramic wear layer mix in the process of snowfall, the initial effect of snow melting is not very apparent, but taking into account the actual road process, under the action of vehicle load, salt will be dissolved into water and the snow-melting effect will improve. In the comprehensive snowfall snow-melting test, it can be concluded that, when mixed, the salt-storing ceramics wear layer mix has a certain snow-melting effect, and at dosages of 40% or higher, the salt-storing ceramic wear layer mix has a more significant ice- and snow-melting effect.

### 2.3. Infrared Spectral Test Analysis of Salt-Storing Ceramic Granules

**Figure 11:**
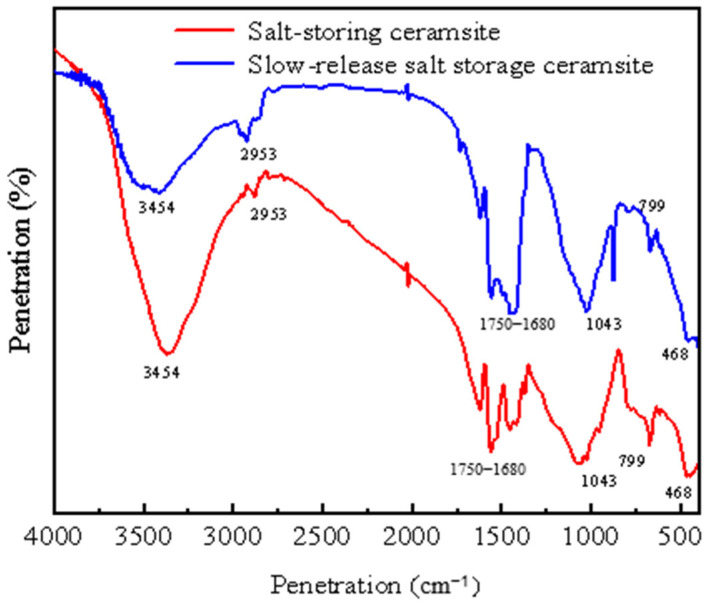
Infrared spectrum of salt-storing ceramsite.

Analyzing Figure 11, it can be seen that the infrared spectra of salt-storing ceramic granules before and after being wrapped with polymer-modified cement mortar (slow-release salt-storing ceramic granules) showed an insignificant change in the positions of the wave peaks, but there was a significant change in the areas of the absorption peaks associated with different functional groups [39,40]. The strong and wide absorption peak at wave number 3454 cm^−1^ indicates the presence of an -OH absorption peak in the conjugated state; the absorption peak at 750~700 cm^−1^ indicates that there is a C-Cl group, and the absorption peak at 468 cm^−1^ is determined to be the absorption peak of sodium chloride, indicating that sodium chloride and calcium chloride, the primary snowmelt salts, have adhered to the shale terracotta grains; and the presence of a carboxylate group can be determined by the presence of a C=O bond at 1750~1680, which shows that sodium acetate and calcium acetate have adhered to the shale grains. At the microscopic level, this demonstrates the successful ceramic salt adsorption of snowmelt salts. Located at 2935 cm^−1^, 2869 cm^−1^, and 1741 cm^−1^ are three absorption peaks with significantly increased vibration intensities, reflecting the methyl (-CH3-) and methylene (-CH2-) groups, and -C=O increased the number of -CH3- and -CH2- groups, which are often found in a variety of organic compounds and can be introduced into the auxiliary alcohol ester twelve, as well as gel-based silica-propylene emulsion doping caused by the increase in the number of functional groups. The number of functional groups increases. The wave number 1625.31 cm^−1^ is the absorption peak of H_2_O, indicating the presence of crystallization water in the material. A clear absorption peak at 799 cm^−1^ is the characteristic absorption peak of the -Si (R) 2O- chain link in organosiloxane, which is the characteristic peak of silicone propylene emulsion. The strong absorption peak at 1043 cm^−1^ corresponds to Si–O–alkyl and Si–O–Si vibrations, which indicates that copolymerization has occurred and suggests that a hydrophobic film has formed due to the action of the silicone–propylene emulsion and the additive alcohol ester XII. This shows that gel-based silicone propylene emulsion completes the hydrophobic modification of cement slurry.

### 2.4. Characterization of Salt-Storing Ceramic Anti-Slip Wear Layers

**Figure 12:**
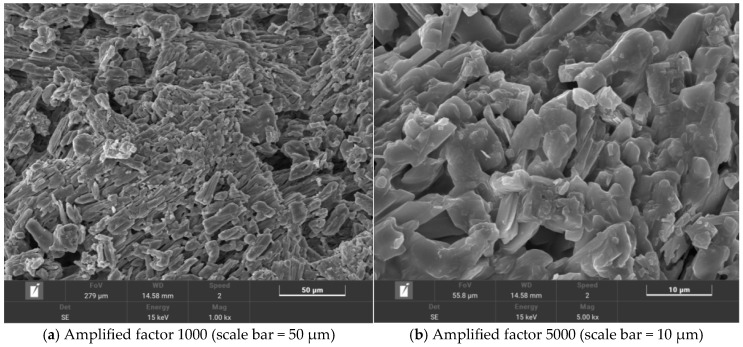
Two-times zoomed salt-storing ceramsite electron microscope diagram.

The micro-morphology of salt-storing ceramic grains is shown in Figure 12.

The observations obtained from the electron microscope image of the salt-storing ceramic grains in Figure 12a (magnified 1000×) show that the snow-melting salt is attached to the pores of the shale ceramic grains in a relatively uniform distribution, and, in Figure 12b, it can be observed that the snow-melting salt is present in the internal pores of the ceramic grains, and the ceramic grains exhibit better adsorption than the snow-melting salt.

**Figure 13:**
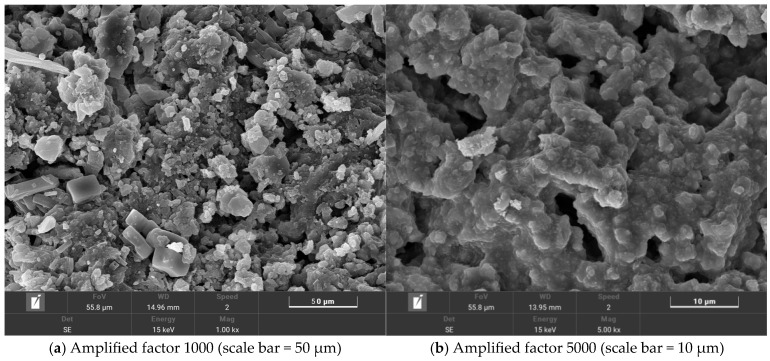
Silicone acrylic emulsion content is 0%.

**Figure 14:**
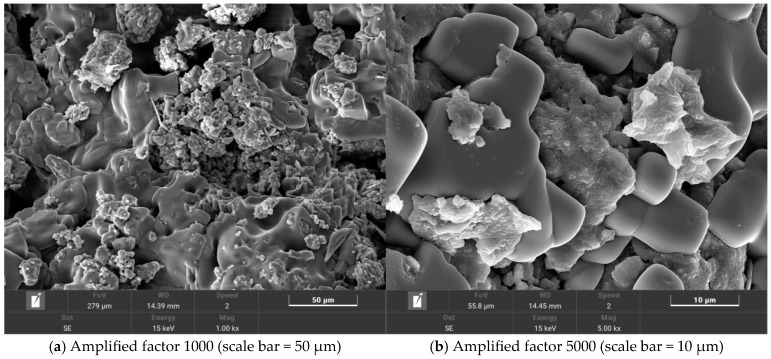
Silicone acrylic emulsion content is 6%.

**Figure 15:**
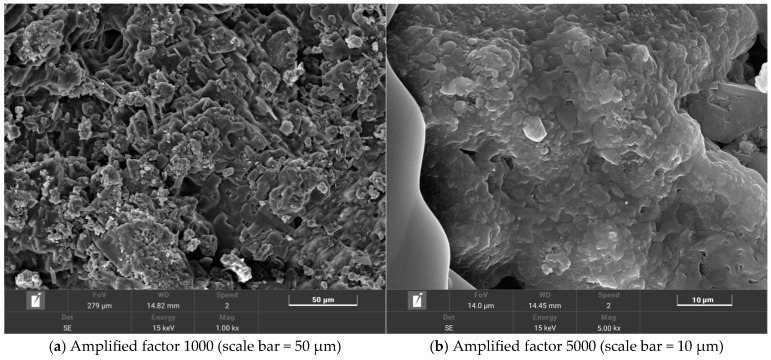
Silicone acrylic emulsion content is 10%.

The microscopic morphology of salt-storing ceramic granules wrapped in ordinary cement mortar (0% gel-based silica–propylene emulsion doping) is shown in Figure 13, and that of salt-storing ceramic granules in 6% and 10% gel-based silica–propylene emulsion-modified cement mortar is shown in Figure 14 and Figure 15.

From the SEM image of ordinary cement mortar on salt-storing ceramic granules in Figure 13, it can be seen that the structure of the cement mortar wrapping layer without gel-based silica–propylene emulsion doping is relatively rough, and there are a large number of pores on the surface of the wrapping layer; combined with the change in conductivity in the slow-release salt storage ceramic granules in the immersion test (Figure 7) of this paper, the salt release of the slow-release salt-storing ceramic granules with 0% of doping silica–propylene emulsion is faster than that of slow-release salt-storing ceramic granule sdoped with silica–propylene emulsion. The surface roughness of the structural form can be judged using SEM morphology, and the existence of many pores reduces the long-term slow-release performance in water-immersion environments, although the ordinary cement-coating layer can serve a slow-release function in the salt release of the salt-storing grains, and the structural layer on the surface of the slow-release salt-storing grains gradually fails after a long period of immersion, resulting in the rapid release of snow-melting salt and an insufficient slow-release performance.

A comparative observation of Figure 14, showing 6% gel-based silica–propylene emulsion doping, shows that, with the doping of gel-based silica–propylene emulsion in the cement mortar, the cement mortar layer begins to form a membrane structure, but, as can be seen from Figure 14a, the membrane system is relatively dispersed, and only part of the area is wrapped by the membrane system. In Figure 14b, magnified 5000×, it can be observed that a small number of pores still exist in the slow-release salt-storing ceramic wrapping layer. This is due to the low dosage of silica–propylene emulsion; the amount of membrane formed by the modified cement mortar is too low, resulting in incomplete encapsulation and residual pores, which results in the slow-release salt-storing ceramic particles not reaching optimal performance.

A comparative observation of Figure 15a and Figure 14a shows that, when the dosage of gel-based silica–propylene emulsion is 10%, it can be found that, after more silica–propylene emulsion is added to the cementitious net mortar, the cement mortar structural layer forms a complete membrane, which essentially encapsulates the salt-storing terracotta grains. Observing Figure 15b, it can be seen that the membrane system’s structure formed on its surface is more dense and uniform. When the dosage of gel-based silica–propylene emulsion reaches 10%, it is able to form a complete membrane structure on the surface of salt-storing grains. Combined with the conductivity test of different dosages of gel-based silica–propylene emulsion-modified salt-storing ceramic granules in Section 2.2 of this paper, it can be concluded that the membrane structure formed by gel-based silica–propylene emulsion-modified cement mortar effectively improves the impermeability and hydrophobicity of cement mortar layer materials, thus realizing the slow release of snow-melting salt components in salt-storing ceramic granules.

**Figure 16:**
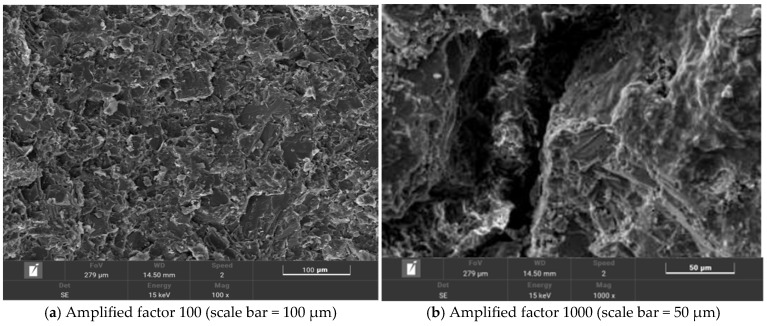
Microscopic topography of salt-storing ceramic-grain-wear layer mixture.

The micro-morphology of the salt-storing ceramic-wear layer mix is shown in Figure 16.

The SMA-10 wear layer mix is formed by the coarse aggregate skeleton, with fine aggregate and asphalt mastic filling the voids, forming a high-density skeleton structure. From the scanning electron microscope image of the salt-storing ceramic grain wear layer mixture in Figure 16a, it can be seen that salt-storing ceramic grains are more uniformly distributed in the mixture, ensuring that the wear layer performs well in melting ice and snow. Voids are present at the mineral–asphalt interface, especially if the bond between ceramic particles and asphalt is not tight and if there is a loose phenomenon. In the 1000× magnified electron microscope image (Figure 16b), it can be clearly seen, in the interface between the mineral material and asphalt transition zone, tha the mineral material surface is bare, and the asphalt shedding phenomenon, visible mineral material, and asphalt mortar bonding force are not enough, and the skeleton still exists in the voids. This indicates that the addition of salt-storing ceramic particles reduces the adhesion between asphalt and mineral material, corresponding to the reduction in road performance of ceramic asphalt mixtures in Section 4.

## 3. Conclusions

(1) In wear layer type selection SMA-10, with salt-storing ceramic particles of the same size and volume blended into the asphalt mixture, salt-storing ceramic particle dosages of 0%, 20%, 40%, 60%, and 80% corresponded to asphalt mixture oil–stone ratios of 6.2%, 6.4%, 6.8%, 7.2%, and 7.5%, corresponding to the asphalt dosage of the segregation loss rate and the rate of loss of fugitive emissions to meet the specification requirements.

(2) Salt-storing grains have different degrees of increase in dynamic stability, showing a trend of initial increase followed by a decrease; when the salt-storing grains dosage is 40%, the dynamic stability reaches the maximum. With increasing salt-storing ceramic grain content, low-temperature performance, water stability, and anti-skid performance weakened to different degrees; when the salt-storing ceramic grain doping is 80%, the water stability of the wear layer no longer meets the specification requirements. In order to ensure that the active slow-release ice- and snow-melting anti-skid wear layer has good road performance, the dosage of salt-storing ceramic grains should be kept below 60%.

(3) The conductivity results of the wear layer mixture show that the abrasive layer does not affect salt precipitation in the salt-storing granules. With increasing salt-storing granule content, conductivity also increases; the value of the conductivity is still guaranteed to have a fast rate of growth, which can ensure the long-lasting effect of the snow-melting effect. Indoor ice-melting and outdoor snow-melting tests show that, with increasing salt-storing ceramic granule content, the snow-melting effect improves, especially at 60% and 80% dosages. Comprehensive road performance and ice-melting performance analysis determined the recommended range of salt-storing ceramic granule mixing to be from 40% to 60%.

(4) The infrared spectra of salt-storing ceramic grains show that the snow-melting salts, sodium chloride and calcium chloride, are attached to the shale ceramic grains, and the ceramic grains are more successful in the adsorption of snow-melting salts. The incorporation of auxiliary alcohol ester XII and gel-based silica–propylene emulsion caused an increase in the number of functional groups, and gel-based silica–propylene emulsion and auxiliary alcohol ester XII formed a hydrophobic film, indicating that the gel-based silica–propylene emulsion completed the hydrophobic modification of the cement mortar.

(5) Through the analysis of the micro-morphological map of the salt-storing ceramic particles and the coating layer, the distribution of snow-melting salts in the pores of the ceramic particles is relatively uniform, and when the dosage of gel-based silica–propylene emulsion reaches 10%, it forms a complete membrane structure on the surface of the salt-storing ceramic particles to realize the slow-release effect of the snow-melting salt components in the salt-storing ceramic particles. Through the analysis of the micromorphological image of the salt-storing ceramic asphalt mixture, it was found that voids exist at the interface between mineral and asphalt; in particular, the ceramic and asphalt bonding is not closed, and there is a loose phenomenon. The incorporation of ceramic particles reduces the bonding force between asphalt minerals, leading to a decrease in the road performance of ceramic asphalt mixtures.

(6) Compared to commercial de-icing methods (e.g., salt-spreading or electrically heated pavements), the gel-modified salt-storing pavement layer delivers persistent snow-melting without external energy, thus reducing salt usage, maintenance costs, and temperature-induced structural stress.

## 4. Materials and Methods

### 4.1. Asphalt and Natural Aggregate

The SBS I-D modified asphalt, provided by Shandong High-speed Construction Materials Co., Ltd. (Jinan, Shandong Province, China), was used in the test, and the basic performance of the modified asphalt was determined according to the ‘Highway Engineering Asphalt and Asphalt Mixture Test Procedures’ (JTG E20-2011) [37]. The technical indicators are listed in Table 3.

The coarse aggregate used in the experimental study was basalt and self-made salt-storing ceramsite (a porous shale aggregate impregnated with de-icing salts) provided by Zhengzhou Zhengfa Municipal Construction Co., Ltd. (Zhengzhou, Henan Province, China), and the filler used was ground limestone powder. According to the specification requirements, the indexes were tested in strict accordance with the ‘Highway Engineering Aggregate Test Procedure’ (JTG E42-2005) [41]. The technical indexes of natural aggregate used in the experimental study are shown in Table 4.

According to the test procedure [42], the basic properties of ore powder were tested, and the results are shown in Table 5.

Fiber plays the role of adsorption, dispersion, stiffening, and toughening in SMA mixtures. In this paper, lignin fiber was selected, and the content was set at 0.4%. The technical properties are shown in Table 6.

### 4.2. Preparation of the Slow-Release Salt-Storing Ceramsite

In this experiment, salt-storing ceramsite was prepared using salt-soaking adsorption. Shale ceramsite with a particle size of 3–5 mm was selected and immersed in a saturated composite snow-melting salt solution at a temperature of 60 °C for 12 h. The optimum content of surfactant PEG-400 was 3%; the present study selected a silicon-acrylic emulsion-based gel material with the best modification effect and determined the silicon–acrylic emulsion dosage in the cement paste to be 10%. The following unified expression is silicone–acrylic emulsion-modified cement paste. The composition and technical properties of the raw materials are shown in Table 7.

First of all, 3–5 mm shale grains were pre-washed and pre-treated to remove the dust and impurities in the pores of the shale grains, which were put into the oven with the temperature adjusted to 100 °C for drying and sparing. Take 500 mL of de-distilled water in a beaker and place it on a magnetic stirrer. The temperature was set to 60 °C; sodium acetate, calcium acetate, sodium chloride, and calcium chloride were added in a 31%:20%:24%:25% ratio and stirred until the solution became saturated; 3% (equivalent to the quality of the ceramic grains) surfactant (PEG-400) was slowly added to the saturated composite snow-melting salt solution, and 500 g of shale grains were then added, soaked for 12 h, removed, and dried for later use.

The specific steps for the slow-release modification of salt-storing ceramic granules are as follows: (1) weigh 400 g of dry salt-storing ceramic granules in a clean tray; (2) mix the polymer emulsions, cement, water, and additives according to specified proportions, and add them to the cement mortar mixing pot and mix, and then mix for 2–3 min to form a cement slurry; (3) add salt-storing ceramic granules to the mixing pot, and then mix for 2 min to perform the slow-release coating of salt-storing ceramic granules; (4) spread the coated granules evenly in a tray and keep for 24 h at room temperature, and then place them in a standard curing environment to complete hardening; and (5) spread the coated salt-storing ceramic granules evenly in a tray and allow them to cure at room temperature for 24 h, and then cure them in a standard environment for 28 days.

In order to determine the optimal dosage of the slow-release treatment agent (silica–propylene emulsion), the test was conducted using modified cement slurry with silica–propylene emulsion dosages of 0%, 6%, 8%, 10%, 12%, and 14% to coat the salt-storing ceramic granules. In total, 400 g of ceramic granules were used, and the cement mass was set to half the mass of the ceramic granules of each group of samples, and the additive used in the silica–propylene emulsion was alcohol ester twelve with a dosage of 6% relative to the emulsion’s solid content; the material dosages used in the test are listed in Table 8.

**Figure 17:**
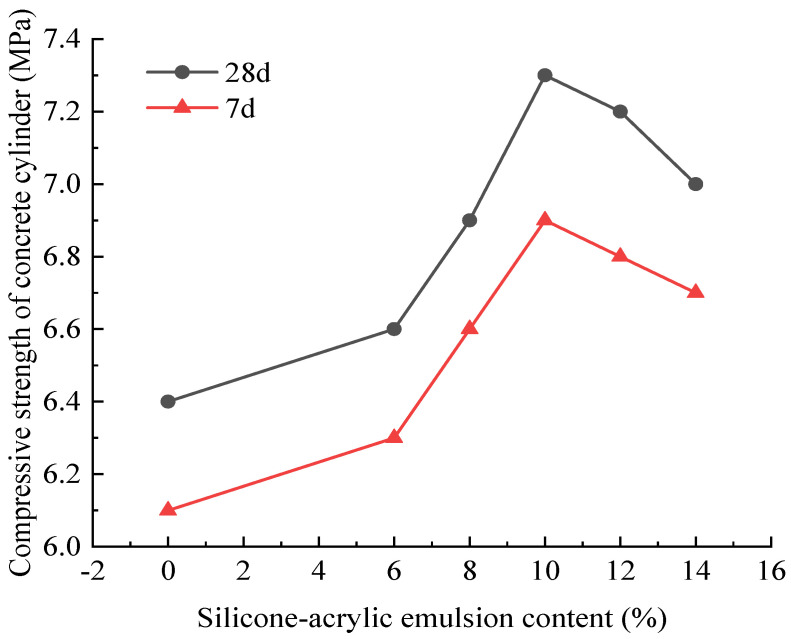
The cylinder compressive strength of salt-storing ceramsite after coating.

Different dosages of silica–propylene emulsion-modified cement mortar significantly wrapped slow-release salt-storing ceramic granules were placed in a standard curing environment for 7 days and 28 days, in reference to the relevant requirements of ‘Lightweight Aggregates and Their Test Methods’ (GB/T 17431.2-2010) [43]. Samples were tested for cylindrical compressive strength after 7 and 28 days of curing; the relationship between silica–propylene emulsion dosage and cylindrical compressive strength of the slow-release salt-storing ceramic granules is shown in Figure 17. 

From the curves in Figure 17, it can be seen that the cylindrical compressive strength of slow-release salt-storing ceramic granules cured for 7 and 28 days shows a trend of an initial increase followed by a decrease with increasing silica–propylene emulsion dosage. The 7-day and 28-day compressive strengths of the test group with silica–propylene emulsion-modified cement mortar are higher than those of the test group without the emulsion, indicating that the silica–propylene emulsion-modified cement coating effectively improves the compressive strength of the ceramic granules with slow-release salt storage. Comparing the 7-day and 28-day results, compressive strength increases with curing time, especially in groups with higher silica–propylene emulsion content. When the silica–propylene emulsion dosage is 10%, the 7-day and 28-day compressive strengths of the salt-storing granules reach their maximum values. The 7-day and 28-day compressive strengths of the salt-storing ceramic granules with a dosage of 10% increased by 13.1% and 14.1%, respectively, compared to the granules wrapped in the 0% (control) group. Therefore, regarding the effect of silica–propylene emulsion-modified cement slurry on the mechanical properties of salt-storing ceramic granules, the optimal dosage of silica–propylene emulsion is 10%.

**Figure 18:**
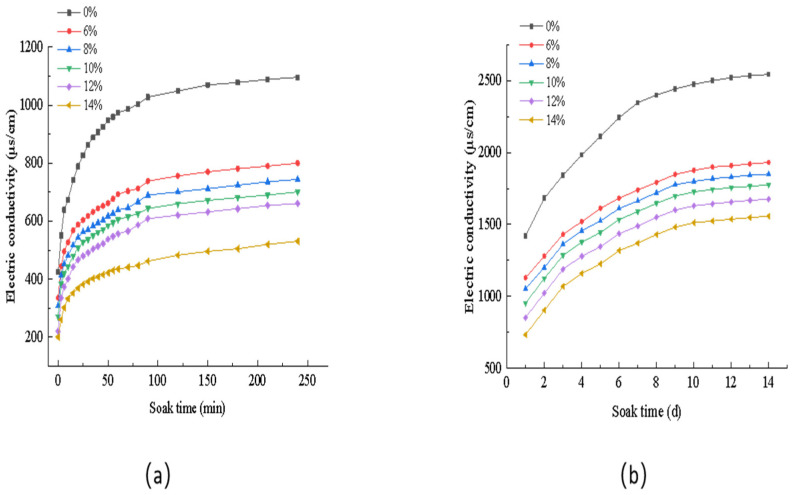
Short-term (**a**) and long-term (**b**) conductivity change in slow-release salt storage ceramsite.

The test was conducted using an immersion dissolution method on granule samples coated with different dosages of silica–propylene emulsion-modified cement mortar; 100 g of slow-release salt-storing granule samples with different dosages of silica–propylene emulsion was taken and immersed in the same mass of water. A DDSA-308A digital conductivity meter was used to record the numerical conversion of the conductivity to measure the short-term conductivity of the salt-storing ceramic granules, and, after 4 h of testing, the conductivity was measured every 24 h to monitor long-term changes in the salt-storing ceramic granules; the results of the short-term conductivity test of the salt-storing ceramic granules are shown in Figure 18a, and the results of the long-term conductivity test are shown in Figure 18b.

Comparing Figure 18a and Figure 18b, by analyzing the short- and long-term conductivity curves of the salt-storing grains, the salt-release rates across test groups with different silica–propylene emulsion dosages are generally consistent, and, after comparing the conductivity trends at different emulsion dosages, it is found that conductivity decreases as silica–propylene emulsion dosage increases, indicating that the silica–propylene emulsion-modified cement mortar acts as a slow-release coating for salt-storing grains, but the dosage of 14% silica-propylene emulsion is slower than that of other dosages of silica-propylene emulsion, indicating that excessive silica–propylene emulsion dosage may hinder salt release from the salt-storing grains.

By observing the conductivity curves for 6%, 8%, 10%, and 12% dosages, it was concluded that the slow-release performance of the salt-storing ceramic granules increased with the increase in the dosage of silica–propylene emulsion in the cement slurry, and the slopes of the long-term and short-term conductivity curves of each group did not show any significant changes in the soaking cycle. In terms of the salt release rate, the higher the dosage of silica–propylene emulsion, the slower the change in the conductivity of the solution, indicating that salt-storing silica–propylene emulsion-modified cement slurry effectively controls salt release from the ceramic granules. Combined with the test of compression strength of salt-storing grains after being coated with silica–propylene emulsion-modified cement slurry, the optimal dosage of silica–propylene emulsion in the cement slurry was determined to be 10%.

### 4.3. Preparation of the Active Slow-Release Melting Ice and Snow Anti-Skid Wear Layer

The test adopts SMA-10 as the wear layer gradation, and a Marshall test was conducted to evaluate the effect of salt-storing ceramic granule dosage on the asphalt aggregate ratio of the wear layer mixture; the results of the oil–rock ratio of each dosing are shown in Table 9.

The optimum asphalt content was verified using the Schellenberg drainage test and the Fort Kentucky fly-away loss test, the results of which are shown in Table 10 and Table 11.

The test results in Table 10 and Table 11 show that the results of the segregation leakage test and fly-away test of salt-accumulating ceramic granules in mixes with different volume dosages meet the requirements of the specification [38].

### 4.4. Road Performance Test

In this test, evaluations of high-temperature stability, low-temperature crack resistance, water stability, anti-slip performance, and permeability were conducted on active slow-release ice- and snow-melting anti-slip wear layers containing different dosages of salt-storing ceramic granules.

(1) Five rutted plate specimens with different salt-storing ceramic granule dosages were molded and held at 60 °C for 4 h during rutting tests to evaluate their high-temperature stability.

(2) Wear layer trabecular specimens were placed in a constant-temperature chamber at −10 ± 0.5 °C for 5 h, and a low-temperature trabecular bending test was conducted using a universal testing machine to evaluate their low-temperature stability performance.

(3) Five types of salt-storing ceramsite asphalt mixtures were formed into two groups of Marshall specimens, with each group consisting of four samples. The first group was immersed in a constant-temperature water tank at 60 °C for 30 min, and the second group was immersed in a constant-temperature water tank at 60 °C for 48 h. The immersion Marshall test was carried out. The test group was vacuum-saturated using a maximum theoretical density device and then placed in a 25 °C water bath for 20 min. The Marshall specimen was loaded into a plastic bag and added to 10 mL of water after sealing. It was placed in a −18 °C low-temperature test chamber for freezing for 17 h. After freezing, it was placed in a 25 °C constant-temperature water bath for 3 h after the stability test. In the control group, a splitting test was performed after soaking at 25 °C for 3 h, and the water stability was comprehensively evaluated.

(4) The sand-paving method was used to determine the construction depth of different salt-storing ceramic granule mixes with different dosages of ice-melting and snow-melting anti-skid wear layer mixes, and the BM-III pendulum friction coefficient tester was used to study the anti-skidding performance of rutted slabs of slow-release salt-storing ceramic granule asphalt mixtures.

(5) Permeability tests were conducted on the rutted asphalt mixture slabs with different dosages of salt-storing ceramic granules to evaluate their permeability performance.

### 4.5. Active Slow-Release Melting Ice and Snow Anti-Skid Wear Layer Melting Ice in the Snow Effect Test

In order to investigate the ice- and snow-melting performance of the slow-release salt-storing ceramic anti-skid wear layer, specimens with different volume fractions (0%, 20%, 40%, 60%, and 80%) of slow-release salt-storing ceramic particles were tested using a wear layer mixture conductivity test, indoor ice-melting test, and outdoor snow-melting test. The test results were used to analyze the effect of different doping levels of slow-release salt-storing ceramic particles on the ice- and snow-melting capacities of the wear layer and road performance to determine the optimal doping of salt-storing ceramic particles on the active melting of ice and snow on the anti-skid wear layer.

#### 4.5.1. Conductivity Test

In this section, Marshall specimens were immersed, and solution conductivity was measured using a conductivity meter; the more snowmelt salt that was dissolved and released per unit time, the higher the conductivity. The salt concentration was estimated by measuring the change in conductivity of the immersed solution, the numerical change in the conductivity meter was continuously recorded during the test, and, after 4 h of testing, the conductivity of the solution supernatant was measured every other day, with theshort-term and long-term conductivity being measured, respectively.

#### 4.5.2. Indoor Ice-Melting Test Trials

Referring to the ice-melting rate test in ‘Ice and snow melting materials for highway asphalt mixture’ (JT/T 1210.2-2018) [38], the ability of salts to melt ice and snow was evaluated. First, Marshall specimens were prepared based on the asphalt mix design. A glass dish with a diameter of 100 mm and a height of 20 mm was filled with water and frozen at a temperature of −18 °C for 24 h to ensure complete freezing of the water. Meanwhile, the Marshall specimens were conditioned in an oven at −5 °C for 4 h. The ice in the glass dish was removed, immediately placed on the surface of the specimen, and then both placed at a constant temperature of −5 °C for 2 h, and the remaining mass of the ice and dish was measured at 10 min intervals. The remaining masses of the specimen types in the test were salt-storing ceramic wearing course asphalt mixtures (SMA-10) with dosages of 0%, 20%, 40%, 60%, and 80%, and two specimens were selected for each group of samples to perform the parallel test.

#### 4.5.3. Outdoor Snow-Melting Tests

Marshall specimens with different dosages of salt-storing ceramic granules were used to simulate road conditions and were placed outdoors during rain and snow conditions to observe their snow-melting effects. Marshall specimens with varying dosages were tested simultaneously for comparison; each group used two specimens, arranged from left to right in the order of 0%, 20%, 40%, 60%, and 80% salt-storing ceramic granule content, to observe the different melting times of the Marshall specimens on top of the snow melting, as well as to analyze the ice- and snow-melting performance.

### 4.6. Infrared Spectroscopy Test

A VERTEX70 Fourier Transform Infrared (FTIR) spectrometer (provided by Shanghai Erdi Instrument Technology Co., Ltd. in Shanghai, China) was used in this experiment. Samples consisted of slow-release salt-storing ceramic granules wrapped with silica–propylene emulsion-modified cement mortar prepared by grinding both types of salt-storing ceramic granules into powder and mixing them with potassium bromide (KBr) in a ratio of 1:100, and then using infrared spectroscopic test presses to prepare presses with good transparency; the infrared spectra were subsequently scanned.

### 4.7. Scanning Electron Microscope Test

In this experiment, a field emission scanning electron microscope (FESEM), model JSM-7500F, was used to observe the microscopic morphology of salt-storing ceramic particles, salt-accumulated ceramic particles wrapped with modified cement mortar with 0%, 6%, and 10% silicone-propylene (SP) emulsion, and abrasive-layer mixtures containing salt-storing ceramic particles. The distribution of snowmelt salt in the ceramic granules, the morphology of the modified cement mortar coating layer with silicone–propylene emulsion, and the microstructure of the salt-storing ceramic asphalt mixtures were analyzed using the obtained micrographs.

## Figures and Tables

**Table 1:** Structural depth test results.

Salt-Storing Ceramsite Content (%)	Paving Diameter (mm)	Structure Depth TD (mm)	Technical Requirement (mm)
0	176.8	1.018	≥0.55
20	179.7	0.986	
40	181.4	0.967	
60	185.0	0.930	
80	186.1	0.919	

**Table 2:** Water seepage test results.

Salt Storage Ceramsite Content (%)	3 Minutes of Water Seepage (mL)	C_w_ Water Permeability Coefficient (mL/min)	Specification Requirement
0	180	40	≤80 mL/min
20	189	43	
40	195	45	
60	204	48	
80	213	51	

**Table 3:** Basic performance specifications of SBS I-D modified asphalt.

Pilot Project	Unit	Test Result
Penetration (25 °C, 100 g, 5 s)	0.1 mm	56
Ductility (5 cm/min, 5 °C)	cm	32
Softening point (TR&B)/°C	°C	78

**Table 4:** Test results of aggregate physical properties.

Performance Index	5~10 mm Crushed Stone	3~5 mm Crushed Stone	3~5 mm Silicone-Acrylic Emulsion Modified Cement Salt Storage Ceramsite	Fine Aggregate
Losangeles wore value (%)	15.1	-	-	-
Apparent specific gravity (g/cm^3^)	2.945	2.932	1.503	2.724
Relative density of bulk volume (g/cm^3^)	2.829	2.818	-	-
Water absorption (%)	0.52	0.55	8.3	-
Compressive strength of concrete cylinder (MPa)	-	-	7.3	-

**Table 5:** Main technical index of mineral powder.

Pilot Project	Unit	Test Result	Standard Requires
Apparent density	t/m^3^	2.726	≥2.50
Moisture content	%	0.2	≤1
Particle size range < 0.6 mm	%	100	100
<0.15 mm	%	96.8	90–100
<0.075 mm	%	86.5	75–100
Hydrophilic coefficient	—	0.56	<1.0

**Table 6:** Technical index of lignin fiber.

Test Items	Unit	Specification Requirement	Test Result	Test Method
Fiber length	mm	≤6	<6	Observation of aqueous solution by microscope
Ash content	%	18 ± 5	18.5	After burning at a high temperature of 590~600 °C, the residue was determined
PH value	—	7.5 ± 1.0	7.7	PH meter measurement
Moisture content	%	≤5	0.3	It was heated in a 105 °C oven for 2 h and then cooled and weighed

**Table 7:** Technical index of raw materials.

Number	Type	Material	Technology Index
1	Gel material	Silicone-acrylic emulsion	Orchid milky liquid, 45% mass fraction of total solid content, PH value: 8.36, glass transition temperature 25 °C, silicone content 11.2%
2	Additives	Alcohol ester twelve	Colorless transparent liquid, no insoluble matter
3	Surfactant	PEG-400	Colorless transparent liquid, PH value: 6.23, viscosity: 83.5 MPa·s
4	Sustained-release materials	Cement	The strength grade is 42.5, the standard consistency water consumption is 25.7, the specific surface area is 343 m^2^/kg, the initial setting time is 177 min, and the final setting time is 239 min

**Table 8:** Different polymer content per modified cement paste ratio.

Polymer Emulsion Content (%)	Quality of Polymer Emulsion (g)	Additive Dosage (g)	Cement (g)	Water (g)
0	0	0	200	80
6	12	0.72	200	80
8	16	0.96	200	80
10	20	1.2	200	80
12	24	1.44	200	80
14	28	1.68	200	80

**Table 9:** Optimum mixture ratio of different ceramide content.

Ceramsite Content (%)	0	20	40	60	80
Optimal asphalt content (%)	6.2	6.4	6.8	7.2	7.5

**Table 10:** Leakage test results.

Salt Storage Ceramsite Content (%)	Technology Index	Test Result	Test Method
0	≤0.1	0.045	T 0732
20		0.050	
40		0.053	
60		0.059	
80		0.068	

**Table 11:** Flying test results.

Salt Storage Ceramsite Content (%)	Technology Index	Test Result	Test Method
0	≤15	4.3	T 0733
20		6.8	
40		7.5	
60		9.3	
80		11.5	

## Data Availability

The authors confirm that the data supporting the findings of this study are available within the article.
